# Systemic effects of starved fibroblast culture supernatant on immunosuppressed rats treated with cancer stem cells (LA7)

**DOI:** 10.22088/cjim.11.2.135

**Published:** 2020

**Authors:** Roghayeh Pourbagher, Farideh Feizi, Haleh Akhavan niaki, Davood Sabour, Ebrahim Zabihi, Hossein Ghorbani, Sahar Gooran, Zeinab Abedian, Fatemeh Majidi, Amrollah Mostafazadeh

**Affiliations:** 1 Student Research Committee, Babol University of Medical Sciences, Babol, Iran; 2 Cellular and Molecular Biology Research Center, Health Research Institute, Babol University of Medical Sciences, Babol, Iran; 3 Department of Pathology of Babol University of Medical Sciences, Babol, Iran; 4 Dental Materials Research Center, Dental Faculty, Babol University of Medical Sciences, Babol, Iran

**Keywords:** Starved fibroblast, LA7, Fibrosis

## Abstract

**Background:**

The present study aimed to investigate and compare the effect of starved

fibroblast culture supernatant (SFS), DMEM and normal saline alone or along with LA7 on dexamethasone-treated immunosuppressed Wistar rats.

**Methods:**

After the isolation of fibroblasts from the fresh foreskin of children, it was cultured in serum-free DMEM, and the supernatant collected after 16 hours (16h-SFS). This solution and the other treatments were injected subcutaneously into the rats from each group once daily for 14 days. The liver, intestine and lung histology along with blood cellular and biochemical characteristics were studied.

**Results:**

The results showed that dexamethasone as immunosuppressant reduced the body weight. The histological change in the liver was mild fibrosis induced by LA7+16h-SFS. Also, among the different blood cellular and biochemical indices measured, the eosinophil percentage in the 16h-SFS treated rats , glucose levels in the 16h-SFS+LA7 group and triglyceride concentrations in the 16h-SFS group were changed (p<0.05).

**Conclusion:**

This study showed that the secretions of starved fibroblasts especially that combined with LA7 cancer stem cells could induce some minor histological and biochemical changes in immunosuppressed rats, and also it opened a new window for subsequent investigations on unknown mechanisms related to this work.

Tumorigenesis is a (multi) complex biological process in which different types of cells contribute in an orchestral manner (1). This cellular network consists of malignant cells, endothelial cells, immune cells, and fibroblasts, among of which non-tumor cells play different roles but orchestra to angiogenesis and tumor metastasis, and fibroblasts of stromal cells play an important role in angiogenesis (cancer-associated fibroblast) (1, 2). In addition to collagen synthesis, fibroblasts have secretory phenotype releasing different compounds into the extracellular matrix, each of which can have important roles at all stages of cancer progression, wound healing and regeneration (2). Cancer stem cells are among a few cancer cells surviving during different steps of metastasis and have high potential in initiating and promoting metastasis (3). LA7 with especially expressed markers shows high tumorigenicity characteristics and is considered as a reliable model for studying the development and differentiation of breast cancer (4). Research about metastatic breast cancer usually needs the methods for transduction and the evolution of tumor in animal models. Different methods have been introduced in this case, and the induction of breast cancer tumor by injection of LA7 cells cultured in the medium is a relatively new method applied in cancer research in vivo (4, 5). The tumors with a size less than 100 μm seemingly lack vessel, and it can be said that such tumors suffer from nutrition and oxygen shortage (like serum starvation).

Thus, angiogenesis plays a pivotal role in tumor survival and promotion (6). Indeed, serum starvation nearly is considered as a model for a biological study on tumor (7, 8). We have already reported that in serum starvation, fibroblasts secrete a dozen proteins to accelerate wound healing and the angiogenesis in Wistar rats (9, patent: H01G:A61B-2014). This solution induces a rate of proliferation in fibroblasts after return to normal culture system (with serum). We expect that these proteins should have some positive effect through angiogenesis on tumor development or progression and spreading (metastasis). 

In this study, we used LA7 as a model to examine this hypothesis. Because LA7 originates from Sprague Dawley rat, we initially used dexamethasone (3mg/kg) to suppress the immune system and to implant and progress tumor to different organs like liver, intestine, and lung.

## Methods

**Animals: **Thirty female Wistar rats (8-10 weeks old, 244±4.5 g) were provided from the Laboratory Animal Facility at the Department of Medicine, University of Babol, Iran. They were maintained at 25±3°C with relative humidity, a cycle of 12 h-light and 12 h-dark, and standard food pellets and tap water. The rats were divided into 6 groups (A-F) and after acclimation (5 rats per cage), *dexamethasone* was intraperitoneally injected to the animals in all groups (3 mg/kg once daily for 6 days) except group F similarly receiving normal saline (table 1). The weight of rats was measured every 2 days. 

**Cell the line: **LA7 (rat mammary gland tumor cells) were offered kindly by Dr. Davood Sabour and then maintained in Dulbecco’s modified eagle’s medium (DMEM, Biowest, France), supplemented with 10% fetal bovine serum (FBS, Sigma, Germany), 100 µg/mL streptomycin and 100 IU/mL penicillin. All flasks containing these cells were incubated at 37ºC with 5% CO2 saturation. After reaching 90% confluence, the supernatant of cultured LA7 cells was removed from the medium, and the cells were washed with PBS. The cells were detached from the culture flask by adding trypsin (EDTA), put into a falcon tube, centrifuged at 400g for 5 min at 4 °C and washed with PBS. The cells were then counted using a hemocytometer.

**16 hours-starved fibroblast cell culture supernatant: **The isolation of fibroblasts from the fresh foreskin of children (1 to 1.5 months old) was carried out by an enzymatic method using dispase and collagenase enzymes (10). The isolated fibroblasts were incubated in a complete culture medium. After reaching 80% confluence, the fibroblasts were cultured in free-serum DMEM, and the supernatant was collected after 16 hours (16h - SFS) based on our previous report (9).

**Induction of mammary gland tumors: **After six–day *dexamethasone* injection (3 mg/kg), LA7 cell suspensions containing 5×10^6^ cells in 300 μL PBS were injected subcutaneously into the right flank mammary fat pad of each rat from group A (DMEM+LA7), C (16h-SFS+ LA7) and E (Normal saline+lA7) using insulin syringe and a 21-gauge needle; while other groups received DMEM (group B), 16h-SFS (group D). Five rats which were considered as dexamethasone-treated control received only normal saline for indicated days (group F). Then, the special medium for each group, including DMEM (A and B), 16h-SFS (C and D) and normal saline (E and F), were injected into the rats once daily for 14 days (table. 1).

**Table 1:** Experimental design and arrangement of injections

**Groups**	**Medium injection once daily for 14 days (until the 21th day)**	**LA7-PBS injection at the 7th day**	**Dexamethasone injection once daily for 6 days**	**Normal saline ** **injection once daily for 6 days**
A	DMEM	+	+	-
B	DMEM	-	+	-
C	16h-SFS	+	+	-
D	16hSFS	-	+	-
E	Normal saline	+	+	-
F	Normal saline	-	-	+

 **Histological and biochemical examinations: **After 53 days of LA7 cell injection (60^th^ day), the rats were dissected and the liver, small intestine, and lung were removed, fixed in formalin and embedded in paraffin. The blocks were sectioned (5 μm), and stained with hematoxylin & eosin (H & E) or Masson’s trichrome. The stained sections were then evaluated, and images were captured using light microscopy (Olympus BX51, Tokyo, Japan). For biochemical examinations, the blood samples were taken from the tail of the rats after 60 days, centrifuged at 400 g for 10 min and the obtained serum was stored at -80 °C for further use. Different biochemical factors including glucose, urea, triglyceride, alanine aminotransferase (ALT), aspartate aminotransferase (AST), creatinine, albumin, cholesterol, total protein were assessed by commercially available kits (Pars Azmoon Co., Tehran,Iran) using an Auto-analyzer (Response 910, Dia Sys, Holzheim, Germany). 

Also, some cellular characteristic of blood samples including the percentage of white and red blood cells, neutrophil, eosinophil, lymphocyte, platelets, and monocytes were measured. 

**Statistical analysis: **The data were expressed as mean ± SD. The Kolmogorov Smirnov test was used to examine the normality of the data. For multiple comparisons, one- way ANOVA along with Tukey test were used. The significance level assigned was p<0.05. SPSS Version 24 software (IBM corp., USA) was used for statistical analysis. 

## Results

**Fig 1:**
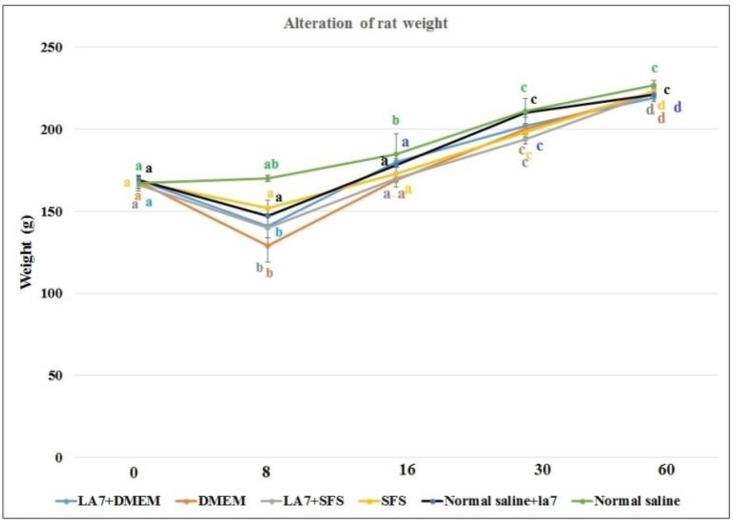
Alterations in the rat weight in different groups at the first day of experiment to the 60th day. Apart from 8th day, the weights of rats increased significantly. Data are shown as mean±SD

To estimate the overall health of the animal, we measured their weight once every two days. 16h-SFS versus DMEM solution and LA7 cells had no significant effect on the rat weight *(*p>0.05). The mean weight of rats in each group increased during the study (fig 1). 

However, compared to group F (as the control group) at the same time, there was a significant decrease in the weight in some groups, especially in the initial days of the experiment. These significant differences were not observed in the second month. Also, there were no significant changes in the weight of liver, lung, and intestine (as a percentage of the organ weight/ total weight) between groups (data not shown).

**Histological findings**: We evaluated the histopathological changes in the lung and liver qualitatively and semi-quantitatively, and determined some hematological indices implying the bone marrow function. 

**Fig. 2:**
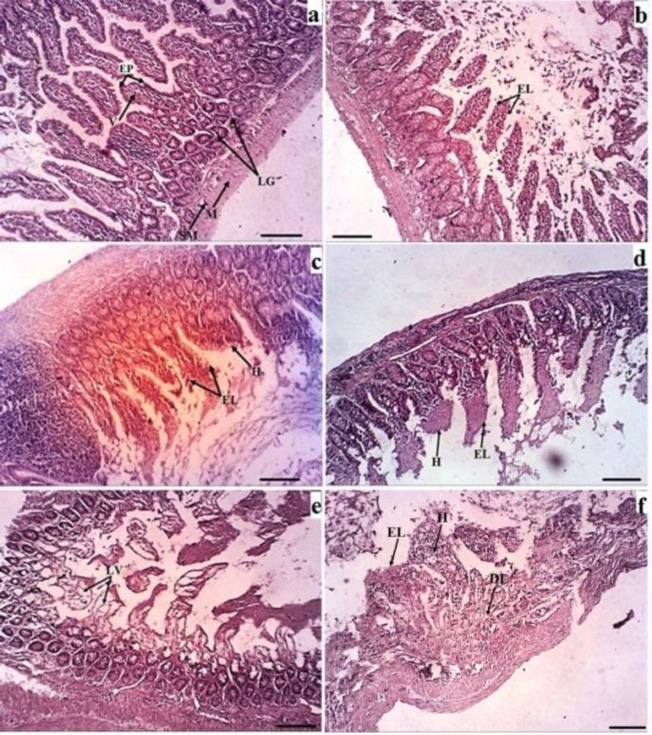
Histopathology of the intestine stained with H&E in the different groups. (a) (Normal saline): EP (Epithelium), LG (Lieberkuhn's gland), M (muscle), SM (submucosal). (b) (Normal saline + LA7): EL (epithelium loss),). (c) (DMEM): EL (epithelium loss), (H (villous edema). (d) (LA7+DMEM):, EL (epithelium loss), H (villous edema), (e) (LA7+16SFS):, loss of villi. (f) (16h-SFS): EL (epithelium loss), H (villious edema, Scale: 2oo µm, ×29. The most abundant damage was observed in 16h- SFS group

Moreover, because of important roles of the intestine in the absorption of the different types of nutrients and subsequently in health, we also studied the intestine microscopically. In comparison with the control group, all treated groups showed some degrees of histopathological changes in the intestine in which these changes varied maximally in 16h-SFS treated rats and minimally in LA7 + normal saline treated animals (fig. 2 and table 2). 

**Table 2:** Histological damages observed in the liver, intestine and lung from the different groups. Lesions were scored based on their severity, according to the method described by Klopfleisch (2013). None (-). Mild (+), moderate (++), severe (+++) (11)

**Study Groups**						**Parameters**	**Organs**
**Normal Saline**	**LA7+ Normal saline**	**16h-SFS**	**LA7+ 16h-SFS**	**DMEM**	**LA7+ DMEM**		
**-**	-	-	+	-	-	Fibrosis	Liver
**-**	-	+	+	-	-	Inflammation	
**-**	+	+	+	+	+	Pyknosis	
**-**	+	+	++	+	++	Congestion of vessel	
**-**	-	-	-	-	-	Necrosis	
**-**	-	-	-	-	-	Submucosal necrosis	Intestine
**-**	-	-	-	-	-	Crypt necrosis	
**-**	-	+++	+	+++	++	Loss of villous tissue	
**-**	-	-	-	-	-	Necrosis of villi	
**-**	+	+++	+++	++	+++	Epithelium loss at villi	
**-**	-	+++	+	++	+++	Villous edema	
**-**	-	-	-	-	-	Fibrosis	Lung
**-**	+	++	+	+	++	Inflammation	
**-**	+	-	+	-	+	Foam cells	
**-**	+	+	+	+	+	Congestion	
**-**	+	++	+	+	+	Damage to alveolus	

**Fig. 3:**
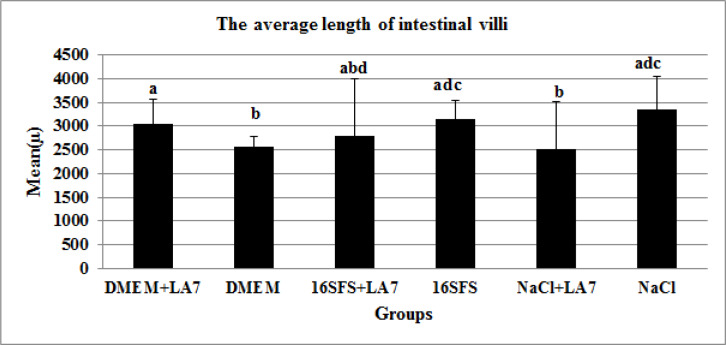
The average length of intestinal villi in different groups measured by Motic images

Although, the mean of the villi length in the latter group was less than that in the 16h-SFS treated group. Surprisingly, the starved fibroblast supernatant treated rats had significantly longer villi compared to DMEM treated group with no significant difference to control group (fig. 3). 

Plus 2.0 ML. The most abundant change in the length of villi compared to normal saline group was observed in normal saline + LA7 group. Data are shown as mean±SD.

**Fig. 4:**
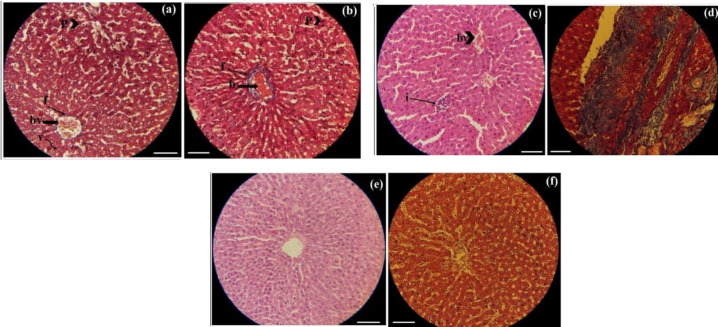
Histopathology of the liver stained with H&E and Masson’s trichrome in the different groups. (a) (DMEM +LA7; Masson’s trichrome): p (pyknosis), v (vacuoles), bv (congestion). (b) (16h-SFS +LA7; Masson’s trichrome): p (pyknosis), f (fibrosis), b (congestion in and around the central vein. (c) (16h-SFS; H&E): i (inflammation), bv (hyperemia). (d) (16h-SFS; Masson’s trichrome):. (e) (Normal saline; H&E): without damage. (f) (Normal saline; Masson’s trichrome): without damage. Scale: 2oo µ, Zoom: ×29. Mild fibrosis was observed only in the liver of la7+16h-SFS treated rats

**Fig. 5:**
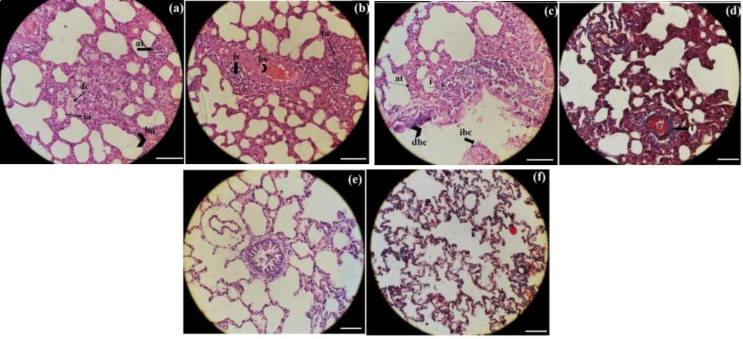
Histopathology of the lung stained with H&E and Masson’s trichrome in the different groups. (a) (DMEM+La7; H&E): at (alveolar wall thickening), fc (foam cell), ia (inflammation of alveolar wall), ba (congestion in alveolar vessels). (b) (16h-SFS; H&E): ta (thickening of alveolar wall), bv (venous congestion), iv (severe inflammation around the vein). (c) (normal saline +LA7; H&E): at (alveolar wall thickening), i (inflammation), dbc (degeneration of bronchial wall), ibc (inflammatory cells in bronchial secretion). (d) (normal saline +LA7; Masson’s trichrome):. (e) (normal saline; H&E): normal structure). (f) (Normal saline; Masson’s trichrome): almost without fibrosis. Scale: 2oo µm, ×29

**Blood biochemical and cellular characteristics: **In addition to the structural study of the indicated organs, we investigated the function of these organs through the assessment of some important blood analytes such as total protein, urea, glucose, creatinine, triglyceride, albumin, AST, ALT and cholesterol. 16h-SFS and LA7+16h-SFS increased serum triglyceride, glucose, respectively, and LA7+DMEM decreased glucose level. There was no significant difference between dexamethasone-treated and –untreated animals in other indicated analytes. We were not able to see any significant difference in the absolute count of red blood cells, white blood cells and platelets, monocytes, neutrophil and leucocyte between dexamethasone-treated animals and untreated groups after 60 days. Nevertheless, the percentage of eosinophil was significantly higher in the 16h-SFS treated group than DMEM, DMEM+LA7, and 16h-SFS+LA7.

**Fig. 6:**
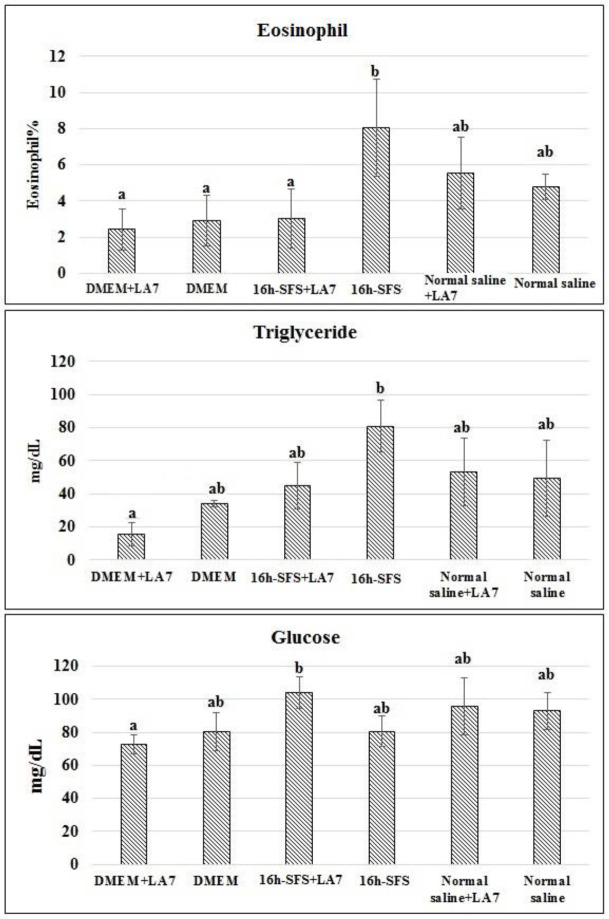
Blood biochemical and cellular alterations in the different groups after 2 months treatment with indicated solutions. The different hematological and biochemical characteristics measured, the glucose, percentage of eosinophil and triglyceride had significant differences among groups. In comparison to vehicle medium (DMEM), 16h-SFS solution was able to increase glucose level in LA7 injected rats. The different letters show that there was a significant difference between groups, with p<0.05 was considered as significant

## Discussion

In the present research, the systemic effects of starved fibroblasts culture supernatant with or without La7 cell line in dexamethasone-treated Wistar rats were studied. Compared to normal saline injected rats, the most important finding was a negative trend of weight in dexamethasone-treated groups and significant decrease on the 8^th^ day, despite the current idea implying that corticosteroid-related drugs administration causing obesity. 

Although dexamethasone had a significant effect on the weight of rat, there is no report to show histological damages of dexamethasone in the liver, lung, and intestine of rat after intraperitoneal exposure, and some studies introduced dexamethasone as a therapeutic and protective agent (12-14). However, Kumar et al. showed that dexamethasone could cause hepatic steatosis in the rat (15). Nevertheless, the results of the present study showed that pyknosis occurred in the liver cells of all groups treated with dexamethasone. Pyknosis shows stress condition (e.g., oxidative stress) and occurs in dying cells due to nuclear condensation (16, 17).

Another finding obtained in this study was the mild fibrosis in liver of rats treated with LA7+16h-SFS.This fibrosis was not observed in other groups including 16h-SFS group. It means that the tumor cells and 16h-SFS solution may have synergistic effect in this observation. As our knowledge, there is no report in literature indicating that the LA7 ability in induction liver fibrosis in rat. However, it seems that starved fibroblasts secrete some compounds that induce these damages. As we know, fibrosis is an excessive production of extracellular matrix especially collagen by fibroblasts, due to some immune signals, myofibroblast factors and special molecular pattern produced by pathogenic organisms (18). In other words, fibrosis is a wound with continuous and excessive repair activation (2). A previous study showed that starved human fibroblasts secrete some acidic proteins which can induce repair activation in the wound healing process (9). It seems that these proteins may contribute to fibrosis occurrence in the liver of rats receiving LA7. Some organs have more potential to be affected by fibrosis including lung, liver, kidney, and heart (19) in agreement with the present study. Another definition of fibrosis can explain this situation: fibrosis is the disruption of extracellular matrix homeostasis due to the excessive production of some components especially collagen tumor cells like LA7 can disrupt the homeostasis through activation of fibroblasts and some immune cells resulting in fibrosis; and this event leads to enhancing the potential of tumor cells to be colonized (19-21). 

Histological study in the three organs (table 2) indicates that the occurrence of inflammation was more than fibrosis Previous studies showed that the occurrence of inflammation in tissue is a stimulator for fibrosis (22). Inflammation in the rats treated with LA7+16h-SFS and 16h-SFS was slightly more than the other groups. Nevertheless, cellular blood characteristics showed that the percentage eosinophil only in 16h-SFS treated rats had a significant difference with other groups. Although eosinophil promotes inflammation through IL-4 and TGF-β, (22) the result of this study did not show this connection; and it seems that more percentage of eosinophil in the 16h-SFS group is due to more inflammation compared to the other groups. In response to inflammation, eosinophil migrates from bone marrow to the affected sites to maintain homeostasis (23). According to the present results, it seems that foam cell formation in the lung is related to the LA7, and all groups treated with LA7 showed this damage in this organ, and probably these two cells can be related to each other by fibrosis. Nevertheless, there is no report to explain the relationship between cancer stem cells and foam cell formation (24). 

Also, diverse factors including IL33 (25), glucose concentration (26), αVβ3 integrin (27), heregulin-β1 (28), peroxisome proliferator-activated receptor γ (PPAR γ), (29) fibrosis and complex interactions among them, can suppress or induce foam cell formation (30) According to the results of this study, despite the measurement of different biochemical indices, only serum triglyceride, and glucose concentration had a significant difference between some groups. Li et al. (26) showed that high glucose concentration could induce foam cell formation through lectin-like oxidized LDL receptor-1 (LOX-1) route. Comparison of glucose and foam cell data in figure 6 and table 2 showed that the most concentration of glucose (p<0.05) was observed in group C (16h- SFS+LA7) with foam cell formation. Some incoordination between glucose concentration and foam cell formation can be related to other factors affecting foam cell formation cited in other works (24-30). Foam cells are common histological damages in lung fibrosis (30) and, however, our results did not show any relationship between fibrosis and foam cell formation, probably due to interactions of the other factors. Foam cell formation may be one reason for alteration of triglyceride concentration. Although the involvement of intestine as a metastatic site of breast cancer is rare but possible, and lower than 1% of previous studies on breast cancer metastasis showed this situation (31, 32). We were not able to observe any tumor cell in this organ. 

In conclusion collectively, this study showed that starved fibroblasts culture supernatant (16h-SFS) with or without LA7 could induce some degree of histological changes and alterations in a few blood cellular and biochemical indices in dexamethasone-treated Wistar rats, so some of these changes were more severe with LA7. Mild fibrosis was one of the damage in the liver treated with LA7+16h-SFS. This study opened a new window for subsequent investigations that will clarify other underlying mechanisms related to this work and took a positive step in breast cancer research.
